# Comparative genomics of mutualistic viruses of *Glyptapanteles *parasitic wasps

**DOI:** 10.1186/gb-2008-9-12-r183

**Published:** 2008-12-30

**Authors:** Christopher A Desjardins, Dawn E Gundersen-Rindal, Jessica B Hostetler, Luke J Tallon, Douglas W Fadrosh, Roger W Fuester, Monica J Pedroni, Brian J Haas, Michael C Schatz, Kristine M Jones, Jonathan Crabtree, Heather Forberger, Vishvanath Nene

**Affiliations:** 1 J Craig Venter Institute, Rockville, MD 20850, USA; 2 USDA-ARS Invasive Insect Biocontrol and Behavior Laboratory, Beltsville, MD 20705, USA; 3 USDA-ARS Beneficial Insect Introduction Research Laboratory, Newark, DE 19713, USA; 4 Center for Bioinformatics and Computational Biology, University of Maryland, College Park, MD 20742, USA; 5 Institute for Genome Sciences and Department of Microbiology and Immunology, University of Maryland School of Medicine, Baltimore, MD 21201, USA; 6 Current address: Department of Biology, University of Rochester, Rochester, NY 14627, USA; 7 Current address: Institute for Genome Sciences, University of Maryland School of Medicine, Baltimore, MD 21201, USA; 8 Current address: J Craig Venter Institute, La Jolla, CA 92121, USA; 9 Current address: Department of Veterinary Microbiology and Pathology, Washington State University, WA 99164, USA; 10 Current address: The Broad Institute, Cambridge, MA 02142, USA

## Abstract

**Background:**

Polydnaviruses, double-stranded DNA viruses with segmented genomes, have evolved as obligate endosymbionts of parasitoid wasps. Virus particles are replication deficient and produced by female wasps from proviral sequences integrated into the wasp genome. These particles are co-injected with eggs into caterpillar hosts, where viral gene expression facilitates parasitoid survival and, thereby, survival of proviral DNA. Here we characterize and compare the encapsidated viral genome sequences of bracoviruses in the family Polydnaviridae associated with *Glyptapanteles *gypsy moth parasitoids, along with near complete proviral sequences from which both viral genomes are derived.

**Results:**

The encapsidated *Glyptapanteles indiensis *and *Glyptapanteles flavicoxis *bracoviral genomes, each composed of 29 different size segments, total approximately 517 and 594 kbp, respectively. They are generated from a minimum of seven distinct loci in the wasp genome. Annotation of these sequences revealed numerous novel features for polydnaviruses, including insect-like sugar transporter genes and transposable elements. Evolutionary analyses suggest that positive selection is widespread among bracoviral genes.

**Conclusions:**

The structure and organization of *G. indiensis *and *G. flavicoxis *bracovirus proviral segments as multiple loci containing one to many viral segments, flanked and separated by wasp gene-encoding DNA, is confirmed. Rapid evolution of bracovirus genes supports the hypothesis of bracovirus genes in an 'arms race' between bracovirus and caterpillar. Phylogenetic analyses of the bracoviral genes encoding sugar transporters provides the first robust evidence of a wasp origin for some polydnavirus genes. We hypothesize transposable elements, such as those described here, could facilitate transfer of genes between proviral segments and host DNA.

## Abstract 2

Comparative genome analysis of two endosymbiotic polydnaviruses from Glyptapanteles parasitic wasps reveals new insights into the evolutionary arms race between host and parasite.

## Background

The capacity to sequence and analyze complex genomes has enabled rapid progress toward understanding atypical biological systems, such as the obligate mutualistic association of polydnaviruses (PDVs) with certain parasitic wasps [1]. PDVs have evolved a distinctive life strategy as they exist in two distinct forms, as a proviral form integrated into the genome of male and female parasitoids [2,3], and in a virus form that is replication deficient. PDV encapsidated genomes are unlike any other viral genomes as they consist of multiple circular double-stranded DNA molecules, referred to as segments. Proviral DNA is amplified from the parasitoid genome, followed by excision, circularization, and encapsidation of segments into virus particles, and occurs only within female ovarian calyx epithelial cells [4-6]. PDVs are also distinctive in that they require two separate hosts to maintain their life cycle. PDV virions are released into the oviduct lumen with no obvious pathology to the primary host. During oviposition, virions, wasp eggs and other parasitism-associated factors are delivered into a secondary host, usually a lepidopteran, where PDV gene expression disrupts immune functions, physiology, and development [7-9]. Virion-induced pathology within the secondary host ensures survival of the PDV proviral form within the parasitoid life cycle.

Genome sequencing and analyses undertaken to elucidate the genetic complement of PDVs relating to their unusual biology have focused mainly on encapsidated viral genomes as isolated from female parasitoid reproductive tracts. Comprehensive genome studies have been conducted for representative members of the two genera of PDVs, bracoviruses (BVs) and ichnoviruses (IVs), described as obligate endosymbionts of braconid and ichneumonid parasitoids, respectively. Braconid parasitoids harboring BVs are monophyletic and comprise the microgastroid complex of seven subfamilies (Cheloninae, Dirrhoponae, Mendesellinae, Khoikhoiinae, Cardiochilinae, Miracinae and Microgastrinae) with greater than 17,500 species [10]. BV genomes sequenced to date are exclusive to subfamily Microgastrinae, *Cotesia congregata *BV (CcBV) [11], and *Microplitis demolitor *BV (MdBV) [12]; IV genomes sequenced include members from the ichneumonid subfamily Campopleginae, *Campolitis sonorensis *IV (CsIV) [12], *Hyposoter fugitivus *IV and *Tranosema rostrale *IV [13], and from subfamily Banchinae, *Glypta fumiferanae *IV [14]. General characteristics that differ in sequenced encapsidated BV and IV viral genomes include segment number, size and abundance, while similar characteristics include a low gene coding density, multiple intron-containing protein coding genes and the presence of multi-gene families [11-16].

Conventional concepts in virology do not adequately describe the viral and proviral forms of PDV genomes. Encapsidated PDV genomes sequenced to date lack known viral structural proteins and DNA replication-associated enzymes, and there is evidence that in CsIV, a gene coding for one structural protein is not encoded by the encapsidated genome [17]. It has been hypothesized that PDVs have a viral ancestry and have undergone reductive genome evolution, where replication and coat protein genes have been transferred to other regions of the wasp genome [18]. Although PDVs are classified as viruses [1], a contrasting hypothesis is that PDVs have evolved from genetic elements that have captured parasitoid genes and a virion production system [19]. Here, we use the term viral genome to represent segment sequences encapsidated in virus particles and proviral segment to represent the linear integrated form of a circular viral genome segment. We conservatively describe the proviral genome to encompass all proviral segments and their excision motifs.

PDV viral genome sequences have advanced our understanding of PDV-mediated pathology in parasitism and have begun to unravel evolutionary relationships of this unusual group of viruses. However, very little is known about the composition and sequence of PDV proviral genomes beyond the prediction of proviral segment sequences. Early studies based on CsIV showed that several proviral segments were flanked by genomic DNA that was not encapsidated, suggesting CsIV proviral segment sequences are dispersed in the wasp genome [20-22]. In contrast, BV proviral segment sequences are thought to be located at a single locus in a tandem array [23-25]. This hypothesis is based on both *in situ *hybridization evidence where probes from 3 of 30 different CcBV viral segments hybridized to the same region of a single wasp chromosome [25] and studies of CcBV and *Chelonus inanitus *BV (CiBV) in which proviral segments were flanked on one or both sides by a different proviral segment [23,24]. A direct DNA sequence repeat was seen at the boundaries of the few proviral segments examined [23], and it appears to mark the site of proviral segment excision, possibly via conservative site-specific recombination, as a single copy of the repeat was noted within the corresponding circularized viral segment [23]. A working model for BV viral segment production is that they are derived from a large precursor molecule encompassing multiple proviral segments, which is amplified after excision of the precursor from the wasp chromosome [26,27]. Studies of CcBV and CiBV show that there is no replication of mature viral segments [23,26-28].

Recently, we presented a global examination and description of proviral segment sequences of a BV associated with *Glyptapanteles indiensis *(GiBV) [15], a parasitoid of gypsy moth. In contrast to earlier concepts, our data showed that some but not all of approximately 24 GiBV proviral segment sequences are tandemly arrayed. We provided the first detailed analysis of a major part of a PDV proviral genome, a 223 kbp locus labeled proviral locus 1, which encodes a tandem array of 8 GiBV proviral segments separated by inter-segmental regions that varies in length from 117 to 8,369 bp [15]. We also proposed that it was reasonable to consider inter-segmental DNA separating tandemly arrayed proviral segments as components of the proviral genome. Structural and compositional analyses revealed that this array of proviral segments was flanked by 6-7 kbp sequence repeats and that proviral segment sequences had a distinct nucleotide composition from inter-segmental and flanking non-segment DNA. Comparative sequence analysis revealed conserved motifs at the sites of excision of segments from proviral DNA, which suggested that there is directionality to the mechanism of segment excision, a conclusion also supported in CiBV [29]. The motifs we identified are also found in other BV genomes, suggesting a highly conserved mechanism of BV proviral segment excision. Analyses of DNA polymorphisms in the eight GiBV viral segment sequences gave evidence for selection acting on both protein-coding and non-coding DNA, indicating non-coding segmental DNA may serve functional purposes [15].

Building on our previous work, we have expanded the knowledge of the BV proviral form through detailed analysis of an additional five loci encoding GiBV proviral segments. Here we revise our estimate of the GiBV viral segment number to 29, encoded by a minimum of 7 proviral loci, providing a nearly complete description of the GiBV viral genome. In addition, we provide equally extensive data showing similar characteristics of proviral segment sequences of GfBV, a BV associated with another wasp in the same genus as GiBV, *G. flavicoxis*. Comparative analyses reveal a high degree of synteny between loci that code for GiBV and GfBV proviral segments as well as flanking DNA. Not all loci are flanked by sequence repeat elements, but segment excision motifs have been conserved. Proviral segment sequences exhibit clear differences in their nucleotide composition relative to flanking DNA. GiBV and GfBV genes appear to be evolving rapidly, and GiBV and GfBV contain a novel multi-gene family that codes for sugar transporter proteins. We also identify a recent insertion of a transposable element (TE) within a population of GiBV and more ancient TEs associated with BV genomes. This work represents the most comprehensive characterization of PDV proviral genomes and of the structural organization of proviral segments to date.

## Results and discussion

### Sequence characterization of GiBV and GfBV viral genomes

**Figure 1:**
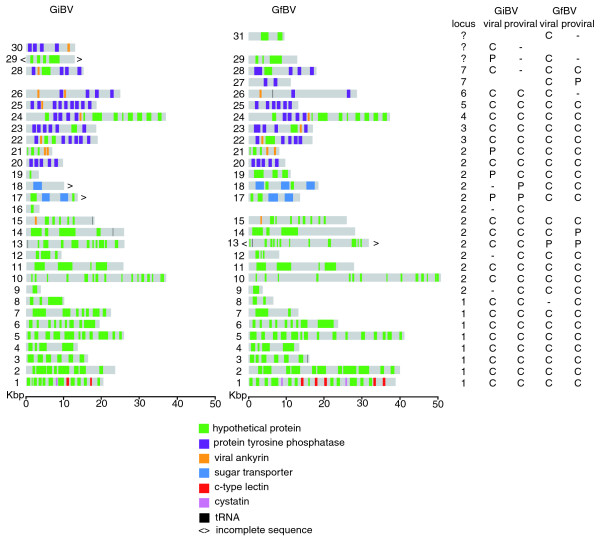
**Summary of GiBV and GfBV sequence data**. Homologous viral segments between GiBV and GfBV are depicted in the same row and have been assigned the same number; blank spaces represent the absence of a homologous segment in one of the genomes. To the right, columns indicate the locus containing the segment and whether the segment is complete (C), partial (P), or absent (-) in the viral and proviral sequences from each genome. Incomplete sequence indicators (<>) are shown only for segments for which both viral and proviral sequence are absent or incomplete.

In an early study, field inversion gel analysis of the GiBV viral genome estimated the presence of 13 segments and a cumulative viral genome size of approximately 250 kbp [30]. At the start of our project the viral genome characteristics of GfBV were unknown, but presumed to be similar to those of GiBV. We undertook a whole genome shotgun (WGS) sequencing approach to sequence the GiBV and GfBV viral genomes. Viral DNA was sequenced to approximately 8× coverage using purified virions pooled from the calyx fluid of approximately 400 and 50 female wasps from *G. indiensis *and *G. flavicoxis*, respectively (see Materials and methods). Following a manual effort to close sequence and physical gaps, we were able to derive a complete consensus viral segment sequence for 21 GiBV viral segments (8 of which were described previously [15]); 4 segments (numbers 17, 19, 21 and 29) remained as partial sequences (Figure 1) due to technical difficulties primarily associated with sequence repeats. GiBV viral segment 25 corresponded to the GiBV genome 'segment F' previously sequenced and characterized in detail [31,32]. For GfBV, we were able to complete sequence analysis of 27 viral segments and 1 (number 13) remained a partial sequence (Figure 1). Nucleotide sequence polymorphisms occurred more frequently in GiBV than in GfBV, and we presume the higher sequence success rate for GfBV was due to sampling a more homogeneous viral population relative to GiBV. The discrepancy in GiBV viral genome statistics with earlier estimates [30] is due to finding multiple GiBV viral segments of similar size, which would have co-migrated on the agarose gel. The 25 GiBV and 28 GfBV viral segment sequences totaled 489 kbp and 581 kbp, respectively. These aggregate genome sizes and viral segment numbers have been further revised based on proviral sequence data (described below). Individual WGS sequence reads were deposited in the NCBI Trace Archive [1643848625-1643870960, 1813616562-1813617310]. Consensus sequence for viral segments, which ranged in length from 9.7 to 39.0 kbp for GiBV and 3.8 to 50.7 kbp for GfBV have been deposited in GenBank [GenBank:EU001243-EU001285].

### Sequence characterization of loci encoding GiBV and GfBV proviral segments

We previously reported that a radioactive probe from total GiBV viral DNA hybridized at varying intensity to 127 clones from a bacterial artificial chromosome (BAC) library of 9,216 clones derived from *G. indiensis *larvae; 17 GiBV viral segment sequences for which probes were available mapped to BAC clones that segregated into 7 distinct non-overlapping groups [15]. A similar size BAC library from *G. flavicoxis *yielded 154 clones that hybridized with total GfBV viral DNA. A combination of BAC end sequencing of 60 BACs and 10 GfBV segment-specific PCRs also segregated these BAC clones into 7 groups. GiBV and GfBV proviral segment sequences not present in the selected BAC clones were isolated by rescreening the arrayed BAC libraries with radioactive viral segment-specific PCR products. Probes for GiBV viral segment number 29 and 30 and GfBV viral segment number 29 and 31 did not hybridize to any BAC clones, while hybridizations of a probe for GiBV segment 28 proved to be false positives. The former may be due to representational bias in the BAC libraries that were screened.

A total of 11 BAC clones from *G. indiensis *(2 BAC clones were from a prior study [15]) and 9 BAC clones from *G. flavicoxis *have been sequenced. Clone selection was based on a combination of proviral segment sequence composition, BAC fingerprinting, and BAC end sequencing to try to ensure that the BAC clones chosen for sequencing contained the greatest possible coverage of proviral segment sequences. The *G. indiensis *and *G*. *flavicoxis *BAC sequence data totaled 1.21 and 1.16 Mbp, respectively. The location of proviral segments within the BAC clones was determined by sequence alignment of viral segment sequences, as well as by searching for individual segment excision motifs (see below). Overlapping BAC sequences were fused, if possible within an inter-segmental region, to create pseudo-molecule sequences reducing unique sequence data for *G. indiensis *and *G. flavicoxis *to 1.08 Mbp and 1.00 Mbp, respectively. The length of contiguous sequences ranged in size from 48.8 to 233.6 kbp for *G. indiensis *and 91.7 to 279.3 kbp for *G. flavicoxis*. Sequences were annotated as described in Materials and methods. These data have been deposited in GenBank [GenBank:EF710644-EF710659; AC191960] was previously submitted [15].

### Identification of novel GiBV and GfBV viral segments

**Figure 2:**
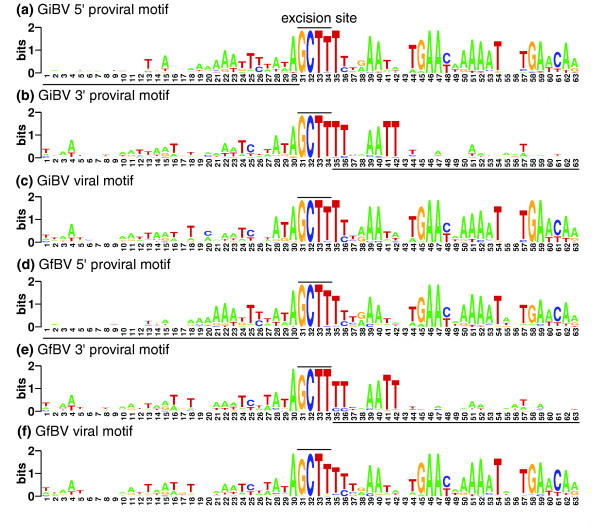
**Nucleotide conservation extends around the proviral segment excision site**. Viral, 5' proviral, and 3' proviral motifs are shown for the GiBV and GfBV genomes, with the excision site highlighted. For each proviral motif, underlined sequence represents non-encapsidated sequence while unmarked sequence represents proviral segment sequence. Following excision, DNA is circularized at the excision site forming the viral motif.

Previously, using MEME [33], we discovered a highly conserved 6 bp DNA sequence embedded within an approximately 60 bp motif that appears to function as the site of excision and circularization of 8 GiBV proviral segment sequences [15]. The analysis of such motifs was expanded to encompass all available viral and proviral sequence data for GiBV and GfBV. Viral, 5' proviral and 3' proviral excision motifs are shown in Figure 2. There are some differences, primarily outside the hexamer repeat, between the new consensus excision motifs for GiBV and those described previously [15]. Additionally, the GfBV excision motif is highly similar to that of GiBV. As we demonstrated before for GiBV, differences between the extended sequence motifs at each end of a GfBV proviral segment indicate there is directionality to the mechanism of segment excision from the site of integration.

Using the extended motifs and MAST [34] we searched BAC sequences in order to detect the boundaries of proviral segment sequences for which we had partial viral segment sequence data, and we extended this search to all BACs. In addition to detecting some of the missing viral segment sequences, MAST predicted the presence of four novel proviral segments for GiBV (numbers 9, 12, 16 and 18) and one (number 8) for GfBV (Figure 1). All these potential proviral segments had exact sequence matches to unassembled sequences in the viral WGS sequence data. Sequence coverage in all cases was below one-fold and viral segment sequence versions of the novel proviral segments could not be assembled from the WGS sequence data alone. To determine whether these sequences represented potential false positives, we searched all inter-segmental and flanking non-segmental regions against the unassembled viral WGS sequence data and found no evidence for them in the WGS data. Thus, a combination of the viral and proviral sequence data predicts that GiBV and GfBV viral genomes each contain 29 segments, although 3 GiBV and 1 GfBV viral segments remain partially sequenced (Figure 1).

### Loci encoding GiBV and GfBV proviral segments exhibit a high degree of synteny

**Figure 3:**
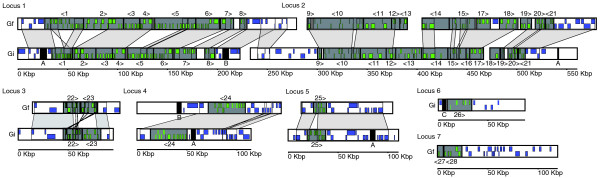
**Structural organization and synteny of proviral segments of GfBV and GiBV**. Detailed diagrams of proviral loci for GfBV and GiBV are depicted. For each, the corresponding segment number of the encapsidated viral segment is given for each segment, with the > or < symbol depicting the directionality of segment excision. Black boxes represent long tandem repeats, and three repeat classes are listed as A, B, and C. Regions of synteny between proviral segment and flanking DNA are shaded in gray. Strand-specific protein coding genes within proviral segments are depicted by green boxes, while genes encoded in flanking DNA at each locus are depicted by purple boxes. The length for each locus is shown in kbp.

Based on GiBV and GfBV proviral segment location and conservation of gene order and sequence similarity within proviral segments, 27 of the 29 proviral segments were classified into pairs of segment homologs (Figures 1 and 3). Each member of a segment pair was assigned the same number. Segments 16 and 30 appear to be unique to GiBV, while segments 27 and 31 appear to be unique to GfBV (Figure 1). In addition to synteny between GiBV and GfBV proviral segments, there is clear evidence for conservation of gene order and identity of genes in wasp DNA flanking proviral segment sequences (Figure 3). These data depict the near entirety of the GiBV and GfBV proviral segments; loci encoding three GiBV and three GfBV proviral segments remain to be identified (Figure 1).

The largest region encoding proviral DNA in both species is represented by two linked proviral loci, 1 and 2. GiBV proviral locus 1 [15] and GfBV locus 1 consist of 8 proviral segments each, while GiBV and GfBV proviral locus 2 consists of 12 and 13 proviral segments, respectively (Table 1). Although the BAC clones we sequenced did not overlap at these loci, consideration of synteny suggests that they are linked (Figure 3). Synthetic oligonucleotide primers were designed near the ends of GiBV proviral segment 17 and 18 to close the existing gap between BAC clones within GiBV locus 2. However, PCR analysis of pupal stage *G. indiensis *parasitoid DNA failed, possibly due the large size of the estimated gap of approximately 20 kbp. Additional primers were designed based on an inter-segmental parasitoid sodium:neurotransmitter symporter gene present in the homologous GfBV region (Figure 3), which were then used in appropriate combination with GiBV proviral segment 17 and 18 primers. An amplicon of approximately 3 kbp (symporter to GiBV proviral segment 18) was obtained and end-sequenced (data not shown), verifying the predicted linkage. We were not able to link the large gap from GiBV proviral segment 17 to the symporter, but the data strongly support linkage of GiBV proviral segment 17 and 18 as occurs in GfBV. Synthetic oligonucleotides designed to either side of the gap separating GiBV proviral locus 1 amplified an approximately 5 kbp product and end-sequence data verified physical linkage of the two loci (data not shown). The strong syntenic relationships between GiBV and GfBV proviral genomes argue for the correct assembly and representation of GiBV proviral locus 2 and its linkage to proviral locus 1 (Figure 3).

**Table 1:** Summary features of loci comprising proviral segments of GiBV and GfBV

	Feature											
	
	Number of proviral segments		Total length (inter-segmental length) in kbp*		Number of predicted genes		% with signal peptides		Median number of exons/gene			
							
Locus	Gi	Gf	Gi	Gf	Gi	Gf	Gi	Gf	Gi	Gf	PTPs	anks
1	8	8	163 (11.7)	202 (9.5)	65	71	79	89	2	2	No	No
2	13	12	219+ (8.9+)	252+ (20.5+)	69	73	12	14	2	2	Yes^†^	Yes^†^
3	2	2	37 (0.3)	34 (0.3)	18	14	0	0	1	1	Yes	Yes
4	1	1	37	37	14	14	0	0	1	1	Yes	Yes
5	1	1	19	13	10	6	0	0	1	1	Yes	Yes
6	1	1^‡^	25	29^2^	6	3	0	0	1	1	Yes	Yes
7	1^‡^	2	15^2^	30 (0.5)	6	8	0	0	1	2	Yes	Yes
Unknown	2	2	26+	22+	9	4	0	50	1	4	Yes	Yes

Total	29	29	541+	619+								

*Measured from the 5' end of the first segment to the 3' end of the last segment. ^†^Locus 2 contains a single segment with protein tyrosine phosphatases (PTPs) and two segments with ankyrins (anks). ^‡^Characteristics of regions of these loci that were not sequenced were hypothesized based on the equivalent locus in the other (GiBV or GfBV) genome.

The remaining GiBV and GfBV proviral loci (Table 1, Figure 3) contain either a single proviral segment (loci 4-6) or two proviral segments in tandem array (loci 3 and 7). Locus 6 and locus 7 sequences are only available for GiBV and GfBV, respectively, but must exist for both genomes based on viral segment sequence data (Figure 1). BAC clones containing loci 3-7 do not overlap with each other or with those encoding loci 1 and 2. The genomic context surrounding three proviral segment sequences from each virus remains unknown, and they could occur as single or tandemly linked sequences. Unless these proviral segments are linked to the loci we have already defined (in which case there would be seven loci), our current data indicate that GiBV and GfBV proviral segment sequences will occupy eight or nine loci. It remains possible that sequence analysis of the missing proviral loci may identify additional viral segment sequences.

We previously described two different long (6-7 kbp) tandem repeats flanking GiBV proviral locus 1 (named L1R1 and L1R2) [15]. These repeats were not found to universally flank the remaining GiBV proviral loci or to flank GfBV proviral locus 1. A long (7.5-8 kbp) tandem repeat similar to L1R1 was found in DNA adjacent to GiBV proviral loci 4 and 5 (Figure 3, A repeats) but these repeats were not found in the homologous GfBV proviral loci. An additional smaller (1 kbp) repeat similar to L1R1 was found on the 3' side of GiBV proviral locus 2. The L1R2 repeat was not found at any other GiBV proviral loci, although L1R2-like repeats (approximately 1.5 kbp) were found near GfBV proviral loci 4 and 7 (Figure 3, B repeats). GiBV proviral locus 6 does have a long (approximately 6 kbp) tandem repeat on the 3' side (Figure 3, C repeat), although it does not share sequence similarity to either L1R1 or L2R2.

As found in GiBV proviral locus 1 [15], inter-segmental regions separating tandemly arrayed proviral segments in both the GiBV and GfBV proviral genomes are generally small (<1 kbp) and do not code for proteins. One exception is the approximately 9 kbp region separating GfBV proviral segments 17 and 18, which codes for a parasitoid sodium:neurotransmitter symporter. As described above, a similar host gene is likely to exist in the homologous region in *G. indiensis*.

### Predicted characteristics of the GiBV and GfBV viral genomes

A summary of the major characteristics of the viral genomes of GiBV and GfBV is given in Table 2, and the segment sequences are visually depicted in Figure 1. These data are based on the combined sequences derived from BV viral and proviral segments, which predicts that both BV viral genomes contain 29 segments, with an aggregated genome size of approximately 503 and 594 kbp for GiBV and GfBV, respectively. GfBV viral segments are, on average, 12% larger than their homologous segment in GiBV, which appears to be due to GfBV-specific tandem duplications of gene clusters. Three GiBV and one GfBV viral segment sequence could not be finished with either viral WGS or BAC sequence data, and assuming a 12% size difference between GiBV and GfBV segment homologs, we estimate that we are missing approximately 14 kbp of GiBV and <1 kbp GfBV viral genome sequence data. We thus estimate a cumulative viral genome size to be 517 kbp for GiBV and 594 kbp for GfBV.

**Table 2:** Genome statistics of BV viral genomes

Feature	GiBV	GfBV	CcBV*	MdBV^†^
Current genome size (kbp)	503	594	568	189
Estimated genome size (kbp)	517	594		
Segments	29	29	30	15
G+C content	36%	35%	34%	34%
Predicted genes	197	193	156	61
Coding density	33%	32%	27%	17%
Predicted tRNAs	3	3	7	7
Proviral loci	7-9^‡^	7-9^‡^	?	?

*Data from [11]. ^†^Data from [12]. ^‡^While seven loci are described here, the two viral segments that were not found in the BAC sequence data are assumed to potentially represent one or two additional loci.

The GiBV and GfBV viral genomes are predicted to encode similar numbers of proteins, 197 versus 193, respectively, and 58% and 63%, respectively, of the genes are predicted to contain introns. Both genomes have a similar average G+C content (35-36%) and protein coding density (32-33%). The genomes contain many gene families found in other bracoviruses, including protein tyrosine phosphatases (PTPs), viral ankyrins, C-type lectins, and cystatins; features of these gene families are listed in Table 3. Encoded PTPs, ankyrins, and cystatins are mostly predicted to be single exon genes while C-type lectins are always predicted to be two-exon genes. PTPs and ankyrins are not predicted to encode signal peptides while C-type lectins and cystatins are often predicted to encode them. Additionally, as reported for CcBV and MdBV, the GfBV and GiBV genomes are predicted to encode a small number of tRNAs (Table 2).

**Table 3:** Features of gene families of GiBV and GfBV

	Feature								
	
	Number of copies			Number with signal peptide		Number of exons		Average	M-K test
						
Gene family	GiBV	GfBV	Loci	Gi	Gf	Gi	Gf	dN/dS (n)	*p*-value
PTPs	42	31	2-7	0	0	1	1-2	0.80 (16)	<0.001
Ankyrins	9	8	2-7	0	0	1	1	0.59 (3)	NA
C-type lectins	2	5	1	1	5	2	2	1.09 (1)	NA
Cystatins	1	2	1	1	2	1	1	2.23 (1)	NA
Sugar transporters	3	5	2	2	0	6-7	7-8	0.23 (2)	NA

NA, not enough data for analysis.

A high degree of partitioning is seen between viral segment gene content when the segments are grouped by proviral locus membership (Table 1). The majority of genes in locus 1 are predicted to encode signal peptides, while few genes in any other loci are. Loci 1 and 2 both contain many proviral segments, most of which encode hypothetical proteins of unknown function and contain one intron. In contrast, the remaining loci contain only one or two proviral segments and predominantly encode PTPs and viral ankyrins that are not predicted to contain introns. A recent study of CiBV showed that genes on the same viral segment were generally expressed in the secondary host in the same time period [35]. Since BV viral segments are present in virion DNA preparations in different abundances, the locus-specific partitioning of GiBV and GfBV proviral segments may represent some additional form of control over BV viral gene delivery.

### Proviral segments and flanking regions show fundamental sequence differences

Annotation of non-encapsidated DNA sequences flanking GiBV and GfBV proviral segment sequences predicted the presence of 78 and 71 genes, respectively. Nine genes that flank GiBV proviral locus 1 were described previously [15]. Very few flanking genes showed similarity to known virus (including PDV) genes; those that did are discussed below. However, many had a high sequence similarity with insect genes (78% for flanking genes and 29% for segment genes; BLASTP, E < e-10). Additionally, flanking genes tended to have more exons per gene than proviral segment genes, although the difference was not statistically significant (2 ± 1 for segment genes and 3 ± 2 for flanking genes).

**Figure 4:**
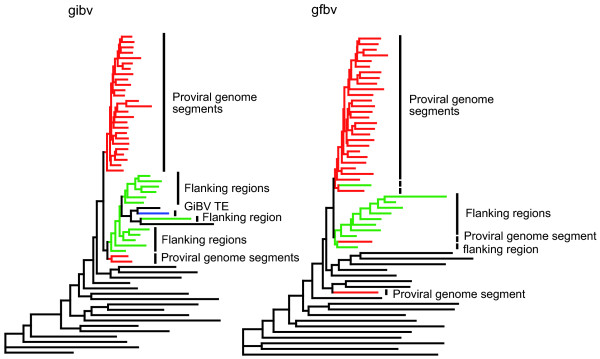
**Clustering diagram of proviral segments, flanking sequences, and inter-segmental sequences by nucleotide composition**. The clustering was generated using the neighbor-joining algorithm on relative trinucleotide frequences for each sequence region. Inter-segmental sequences are shown in black (unlabeled), proviral segment sequences in red, flanking sequences in green, and the GiBV TE in blue.

Proviral segment and flanking DNA are distinct not only at the gene level, but on a nucleotide composition level as well. Previously, we demonstrated that for GiBV proviral locus 1, proviral segment sequences had nucleotide compositions similar to each other and distinct from flanking sequences [15]. We extended the analysis of trinucleotide frequencies to encompass all available GiBV and GfBV proviral segment, flanking, and inter-segmental sequence data. The results are shown in Figure 4. The majority of proviral segment and flanking sequences cluster into distinct groups, and the short terminal-branch lengths indicate highly similar composition within these groups. Inter-segmental regions also tend to cluster together, although with a higher degree of variation, indicated by the longer terminal-branch lengths. This was expected as the generally shorter lengths of inter-segmental sequences make calculating trinucleotide frequencies less accurate. These results were consistent across both the GiBV and GfBV sequence data.

### GiBV and GfBV proviral segment genes are evolving rapidly

As koinobiont endoparasitoids, PDV-carrying wasps develop as larvae within caterpillars that are still undergoing development. Therefore, they are in an arms race with the caterpillars and PDVs contribute to survival against attacks by the immune system of a living caterpillar. Given this situation, it is plausible to hypothesize that PDV genes on a whole must evolve rapidly, particularly since conserved genes that typically control viral replication and particle formation appear to be absent from the viral genome. We utilized sequence divergence between GiBV and GfBV genes to test this hypothesis. All genes described here were divided into two sets: one encompassing genes encoded by proviral segments, and one including flanking genes. Genes were considered orthologs if they had reciprocal best BLAST hits to each other and appeared in syntenic positions. Using these criteria, we identified 72 orthologous gene pairs in the proviral segment gene set and 41 in the flanking gene set.

**Figure 5:**
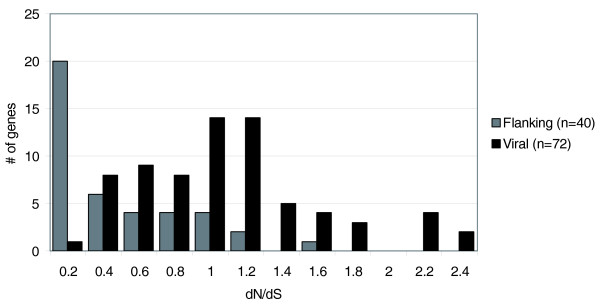
**Histogram of dN/dS values for viral and flanking genes in GiBV and GfBV proviral sequences**. dN/dS values were calculated for 72 and 41 homologous pairs of viral genes in flanking DNA, respectively. Genes in flanking DNA have dN/dS values centered near 0 while viral genes have dN/dS values centered near 1.

In order to examine the strength and direction of selection acting on these genes, the ratio of non-synonymous to synonymous substitutions (dN/dS) was calculated and a histogram summarizing the results is shown in Figure 5. Detailed results for each gene pair are shown in Additional data file 1. Flanking wasp genes predominantly have dN/dS values near 0 (median = 0.23, average = 0.38), indicating that they are under strong negative selection, which is expected for genes involved in essential cellular processes. On the other hand, proviral segment genes have dN/dS values centered near 1 (median = 0.92, average = 0.96), suggesting that many of these genes are under neutral or positive selection. Analysis with the Mann-Whitney U test shows that, overall, segment genes are evolving at significantly different rates than flanking genes (*p *< 0.001).

An alternative explanation for the dN/dS distribution of proviral segment genes centered around 1 could be that we predicted a large number of pseudo-genes, which would be evolving neutrally; this would spike the dN/dS distribution near 1. To test this possibility and more accurately assess positive selection, we conducted a more powerful test of selection, the McDonald-Kreitman (M-K) test [36], by comparing divergence between the 72 orthologous gene pairs to polymorphisms within those genes in the GiBV viral shotgun sequence data. Results of the test (Table 4) show significant evidence for positive selection across GiBV genes as a whole (*p *= 3 × 10^-8^), indicating that most of our gene models are not false predictions and that many GiBV genes are under positive selection. Criticism of the M-K test suggests that a spurious significant result can be produced by purifying selection acting on slightly deleterious mutations within a species rather than positive selection between species [37]. However, it is highly unlikely that slightly deleterious mutations alone could account for the level of significance found in this analysis.

**Table 4:** McDonald-Kreitman test for selection

Differences	S	N	N/S
Polymorphic	240	319	1.33
Fixed	1,472.5	3,262.5	2.22

The number of synonymous (S) and non-synonymous (N) substitutions within GiBV (polymorphic) and between GiBV and GfBV (fixed) are shown. *p *= 3 × 10^-8^, Fisher's exact test.

Closer examination of specific GiBV and GfBV gene families also revealed evidence of potential positive selection, particularly within PTPs, cystatins, and C-type lectins (Table 3). PTPs showed a highly significant pattern of positive selection (*p *< 0.0001) using the M-K test, despite having an average dN/dS below 1. Additionally, the C-type lectin and cystatin analyzed had dN/dS ratios above 1, suggesting that these genes are also under positive selection. While it is plausible that gene families that had uniformly low dN/dS values might be involved in core viral functions such as replication or encapsidation, the only gene family found with such a dN/dS distribution was the sugar transporters (average dN/dS = 0.23). As these genes are specific to GiBV and GfBV and not found in other sequenced BVs, it seems unlikely that they serve a core viral function.

In contrast to the high levels of positive selection detected within proviral segment protein coding sequences, we previously reported that, within GiBV proviral locus 1, non-coding segment DNA appeared to be under purifying selection [15]. However, when the analysis was extended to include all sequence data, non-coding sites were evolving at comparable rates to synonymous sites (data not shown). This suggests that this conservation of non-coding DNA is either restricted to GiBV proviral locus 1 or that previous results were affected by limited sample size.

### A wasp origin of GiBV and GfBV sugar transporter genes

Little is known about the origins of PDV genes. Genes that have sequence similarity to known eukaryotic genes, such as those encoding PTPs, are thought to have eukaryotic origins (see [38] for review). However, these gene families have such diverse sequences within a single PDV genome that phylogenetic analyses have thus far been unable to determine an exact origin of these genes. Recently, in an analysis of genes from previously published BV genomes, Bezier *et al*. [39] found that no BV genes were more similar to their insect homologs than their vertebrate homologs, and phylogenetic analysis did not suggest an insect (or any other) origin for any BV genes.

Annotation of two pairs of GiBV and GfBV segment homologs (segments 17 and 18) revealed a novel family of 8 genes with a surprisingly complex intron-exon structure, consisting of 6-8 exons in each gene as opposed to an average of 2 ± 1 exons in other viral genes. The eight genes are predicted to encode major facilitator superfamily (MFS) transporter proteins, which in general transport a wide variety of small solutes across membranes [40]. The best BLASTP hits for all eight proteins were matches to predicted insect sugar transporters, particularly one from the honey bee *Apis mellifera *and one from the parasitic wasp *Nasonia vitripennis *(approximately 45% amino acid identity to both). Such genes have not been described within PDV genomes sequenced to date.

**Figure 6:**
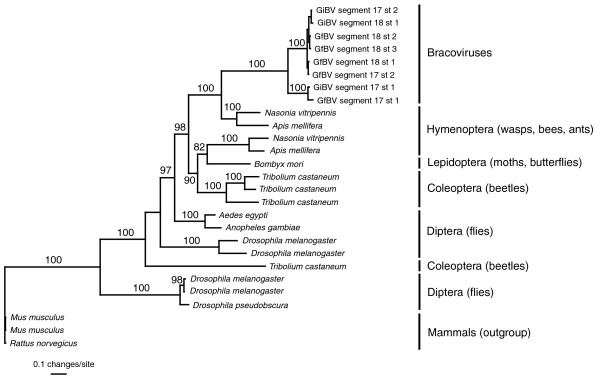
**Bayesian phylogram of sugar transporter gene sequences from GiBV, GfBV and various insects**. Mammal sugar transporters were used as an outgroup. Posterior probabilities >80% are listed above the corresponding branches, and higher taxonomic classification is shown to the right of the tree.

Bayesian phylogenetic analysis using a GTR+gamma+I model was conducted on the 8 GiBV and GiBV sugar transporters and the 16 most similar transporters in insects. Three sugar transporters from vertebrates were chosen as outgroups, as no other arthropod sugar transporters were present in the GenBank protein database. The resulting phylogram is shown in Figure 6, with posterior probabilities given above branches. The GiBV and GfBV sugar transporters group strongly with an orthologous pair of sugar transporters from *Nasonia *and *Apis*, suggesting that these genes share a common hymenopteran ancestry. The sugar transporters did not group with that of the silk moth, *Bombyx mori*, suggesting that these genes were not acquired from the secondary host genome. The similarity of these genes to hymenopteran sugar transporters is likely not due to convergence, as if the genes were acquired from the secondary host genome, they would most likely be expressed in the caterpillar host, and therefore convergence should not drive the genes toward wasp sugar transporters. The low dN/dS values calculated for the sugar transporters (Table 3) suggest that they are under purifying selection. We hypothesize that this, coupled with a recent acquisition event, is why these genes still so closely resemble their ancestral wasp genes. It is unclear why the BV transporters would be under different selective pressures than other described virus gene families such as PTPs. Nevertheless, this finding provides support for the hypothesis that a large number of PDV genes have been acquired from their primary host, but have diverged to such an extent through positive selection that limited similarity remains with their ancestral wasp genes.

### A transposable element in GiBV proviral segment 11

The GiBV proviral locus 2 sequence was generated by merging sequences from overlapping BAC clones. One BAC clone contained an 11,578 bp insertion in proviral segment 11 [GenBank:EU822800], which was not present in the other BAC clone in the region of overlap. The insertion sequence in BAC clone 1C20 is predicted to encode a single gene with sequence similarity to transposase and was bounded by a perfect 8 bp direct repeat with perfect 49 bp inverted repeats internal to the direct repeats, suggesting that the sequence is a TE. Analysis of the transposase using ProteinRepeatMask [41] and CENSOR [42] classified this transposase as *Drosophila *p-element-like. However, the 8 bp direct repeat (AAATTCCA) is different from that typical of p-elements (GGCCAGAC), and the TE is much larger, overall, than typical p-elements, which are only approximately 3 kbp in size. A single copy of the 8 bp direct repeat exists in BAC clone 2C5, which lacks the TE and most likely represents a point of replicative insertion for this class II transposon. The TE insertion does not disrupt a gene. Inclusion of the TE in the nucleotide composition analysis shows that the TE strongly groups with flanking sequences, rather than the segment sequence in which it is inserted (Figure 4).

The TE sequence is not depicted in the proviral locus 2 sequence presented here (Figure 3) as BAC clone 2C5 contained a complete sequence of proviral segment 11 while clone 1C20 did not. In addition, the consensus sequence derived for GiBV viral segment 11 did not contain the TE. A BLAST search of the TE against the unassembled GiBV viral WGS sequence reads revealed four matching reads. These reads matched internal parts of the TE and the junction between one of the TE ends and viral segment 11, suggesting the TE is found in some GiBV viral genomes at a low frequency. We hypothesize that this TE was recently acquired from the wasp genome, and has not yet been lost or gone to fixation in the GiBV genome. If the TE was highly deleterious to the BV genome, it would be unlikely to be present in the population. While this TE does not encode any genes other than a transposase, it does suggest that TEs can enter a BV genome and become packaged successfully. No evidence of this TE was found in either viral or proviral GfBV sequence data.

### CcBV-like genes are linked with GfBV proviral locus 7 and appear to be derived from *Maverick *TEs

**Figure 7:**
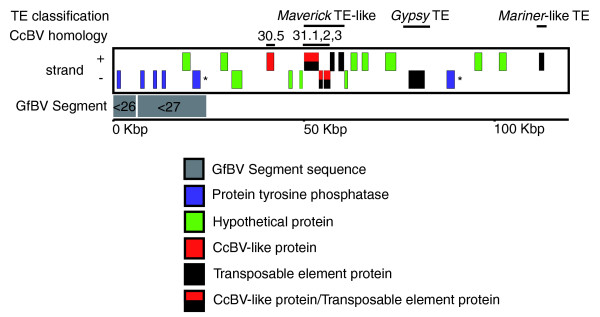
**Detailed view of GfBV proviral locus 7**. Genes are labeled by strand, and genes identical in nucleotide sequences are indicated by asterisks. TE classification and CcBV homologs are shown above the genes. The directionality of segment excision is indicated by an arrowhead next to the segment number.

GfBV proviral locus 7 contains proviral segment 28 and a partial sequence of proviral segment 27, and is flanked on one side by approximately 95 kbp of non-segment DNA (Figures 3 and 7). The latter encodes some common TE elements (*Gypsy *retrotransposon and *Mariner*-like) and, remarkably, genes with sequence similarity to genes identified in the CcBV viral genome (Figure 7). Additionally, this region encodes a PTP gene, which is identical in nucleotide sequence to a PTP gene at the 3' end of GfBV proviral segment 28 (asterisks in Figure 7). MAST searches of this region using the GfBV proviral excision motifs did not reveal any potential excision motifs, and BLAST searches of the region against the unassembled GfBV viral sequence reads did not reveal any matching hits, suggesting that this region does not contain novel GfBV proviral segments.

The genes similar to those in CcBV include one that has strong sequence similarity to CcBV hypothetical protein 30.5 and a block of three genes that are homologous to, and in the same order as, CcBV segment 31 genes 31.1, 31.2, and 31.3 (Figure 7). The gene homologous to CcBV 31.1 contains a DNA Pol B2 domain, which in CcBV was annotated as a pseudogene due to a premature translational stop codon [11,43]. The second and third genes in the block contain an integrase core domain and a poxvirus-like DNA packaging domain, respectively. The CcBV genes were previously considered 'virus-like' and were hypothesized to represent ancestral virus genes within the BV genome [43].

However, ProteinRepeatMasker [41] and CENSOR [42] identified the block of three genes and the next two downstream genes (Figure 7) as components of a *Maverick *TE. *Mavericks *are a novel class of giant TEs [44], also known as *Polintons *[45]. BLAST searches revealed homologs of all five genes to be common components of *Maverick *TEs present in the recently released genome sequence of the parasitic wasp *Nasonia vitripennis*. *Nasonia *belongs to a lineage of wasps separate from those carrying PDVs, suggesting that these proteins are not of BV origin. Based on these results, we hypothesize that the tandem array of three genes in CcBV segment 31 represents remnants of a *Maverick *TE that was transferred to the CcBV proviral genome and has since become fixed.

The *Maverick*-like array of proteins in the GfBV flanking DNA is missing two components common to all *Maverick *TEs: a coiled-coiled domain protein and terminal repeats. The complete absence of terminal repeats coupled with the maintenance of open reading frames is particularly unusual. Since TEs cannot function without terminal repeats, TE open reading frames typically degrade rapidly after their terminal repeats are lost. This suggests that selection on the wasp genome is maintaining these open reading frames, although their potential function is unknown. The abundance of both TEs and BV-like genes encoded in DNA flanking GfBV proviral locus 7 may indicate that this region is a 'hotspot' for the movement of DNA between proviral and flanking sequences. It will be of interest to determine if similar sequences are present in the homologous GiBV locus.

## Conclusion

Here we provide a comprehensive sequence and computational analysis of the viral genome of BVs isolated from two different *Glyptapanteles *species, *G. indiensis *and *G. flavicoxis*, and an analysis of proviral sequences from which encapsidated viral genomes are derived. The sequencing of both BV genomic forms, that is, viral and proviral segments, was critical in developing a more complete definition of both the viral genome as well as the structural organization of a large component of the proviral genome, given that the latter unexpectedly led to the discovery of four GiBV and one GfBV viral segment not evident from sequencing the viral genome.

Although GiBV and GfBV proviral segments are not all tandemly arrayed, synteny between the two sets of sequences and preliminary linkage data for GiBV suggest that approximately 70% of the 29 proviral segments in each genome are clustered in a single genomic region consisting of two proviral loci that span a region of approximately 580 kbp. Each locus contains tandemly arrayed proviral segments separated from each other by a short stretch of non-segment DNA (Figure 3). The remaining GiBV and GfBV proviral loci identified contain either one or two proviral segments. Most of the seven characterized loci are flanked by long stretches of non-segment DNA that encode insect proteins. Not every proviral locus is flanked by long tandem repeats, as was reported for proviral locus 1 [15]. So it is presently unclear how proviral loci are demarcated within the wasp genome sequence. Qualitative and quantitative studies of proviral sequences at the time of production of viral segments will be needed to accurately delineate the boundaries of amplified proviral sequences. Our sequence data are not incompatible with the hypothesis that all proviral loci are linked in a macrolocus, as suggested for CcBV proviral segments [25,27]. Additional mapping experiments, for example, fluorescence *in situ *hybridization analysis using segment-specific probes or generation of a BAC tiling path among loci should resolve the spatial relationships among the GiBV and GfBV proviral loci. If present, a macrolocus encoding proviral segments would exceed 1 Mbp in size.

BVs are thought to have a monophyletic origin about 100 million years ago [46], a hypothesis supported by the high degree of synteny between GiBV and GfBV proviral loci and flanking DNA, as well as the highly conserved proviral segment excision motif among BVs [15]. The compositional difference in sequences at proviral loci could be indicative of independent origins of BVs, but they could also be the result of selective pressures from the primary and or secondary host environment. It will be of interest to compare proviral loci from basal micrograstrines, such as *Microplitis*, to those of more derived miscogastrines such as *Glyptapanteles*. Such comparisons could determine if there was an ancient site for BV integration and, if such a site exists, the extent to which it has been conserved. In addition, whole host genome sequence data are likely to contribute to a more complete understanding of the evolution of BVs.

The functions and origins of PDV genes are often difficult to define. Comparative sequence analyses revealed widespread positive selection across a large number of GiBV and GfBV genes, which supports their role in an 'arms race' between virus and caterpillar. This rapid evolution may help to obscure the origins of many PDV genes. However, GiBV and GfBV both encode a gene family of sugar transporters not found in other sequenced PDV genomes. Phylogenetic analyses suggest that these were wasp genes that were moved into the proviral genome, providing evidence for an insect origin for these BV genes. This suggests that the proviral BV genome may be at least partially derived from the wasp genome, and possibly represents a mosaic of an ancestral virus and more recently inserted host genes.

Transposable elements represent a plausible mechanism for acquiring virulence or other desirable genes from the host [27,47]. Our evidence shows a p-element like and a *Maverick*-like element associated with BV genomes. Whether the latter plays a role in BV biology is unknown, although it is interesting to note the similarities between the proposed methods of replication in *Mavericks *[45] and PDVs, as both are hypothesized to replicate extrachromosomally from a circular or stem-loop molecule. Both described TEs, the former recently acquired and the latter ancient, could have served to move core viral genes out of the ancestral BV genome to the host genome and virulence genes into it. Alternatively, genes could have moved out of the proviral genome by the degradation of excision motifs of proviral segments, thereby creating 'pseudo-segments' that are no longer encapsidated.

## Materials and methods

### Rearing of parasitoid wasps

Outbred populations of *G. indiensis *and *G. flavicoxis*, solitary and gregarious endoparasitoids of gypsy moths (*Lymantria dispar*), respectively, were maintained at the USDA-ARS-Beneficial Insects Introduction Research Unit, Newark, Delaware, until they were moved to Beltsville, Maryland in 2007. The maintenance protocol and history of the *G. indiensis *colony was described previously [15]. *G. flavicoxis *was reared under the same protocol with the exception that *G. flavicoxis *parasitizes late third instar gypsy moth larvae. Cocoons formed from parasitized hosts were stored at 24°C until adult parasitoid emergence and then separated by sex. For BAC library material, *G. flavicoxis *larvae were dissected from parasitized gypsy moth larvae 10 days post-parasitization, briefly rinsed in phosphate buffered saline, flash frozen in liquid nitrogen and stored frozen at -80°C.

### Virion purification and DNA extraction

Virions were purified from *G. indiensis *and *G. flavicoxis *females using established protocols [48]. Briefly, female wasps were anaesthetized in 75% ethanol and rinsed in phosphate buffered saline. Ovaries were dissected from the females in a drop of phosphate buffered saline and ruptured, draining the calyx fluid. Pooled calyx fluid was subsequently filtered through a 0.45 μm filter to remove eggs and cellular debris [49]. Viral DNA was extracted according to established protocols [30].

### Identification of BAC clones containing proviral segment DNA

BAC libraries of *G. indiensis *and *G. flavicoxis *with a 120 kb average insert size were constructed by Amplicon Express (Pullman, WA, USA), using a partial *Bam*HI digest inserted into an *Mbo*I site of a pECBAC1 vector. A nylon filter arrayed with 9,216 BAC clones was created from each library. In order to identify BAC clones containing proviral segment DNA, BV encapsidated viral DNA from each was radioactively labeled with ^32^P-labeled α-dCTP (NEN/Perkin-Elmer, Waltham, MA, USA) using the Redi-prime II DNA labeling kit (Amersham Biosciences, Piscataway, NJ, USA). Labeled DNA was then purified using a QIAquick PCR purification kit (Qiagen, Valencia, CA, USA). The filter was pre-hybridized at 65°C for at least 3 hours with Rapid-hyb Buffer (Amersham Biosciences) and 500 μg of salmon testes DNA (denatured at 100°C; Sigma-Aldritch, St. Louis, MO, USA). The probe was added and allowed to hybridize overnight at 65°C. The filter was then washed 2 times for 60 minutes each at 65°C with a 0.1 × SSC/0.1% SDS solution, wrapped in plastic wrap, and autoradiographed using Kodac BioMax MS film.

### Viral and BAC clone sequencing

Approximately 7.5 μg of BV encapsidated DNA was sheared and DNA fragments in the size range 3.5-4.5 kbp purified after separation by agarose gel electrophoresis. The fragments were blunt ended and, after addition of *Bst*XI adaptors, cloned into the *Bst*XI site of pHOS2. Shotgun libraries were similarly made for each BAC clone. Celera Assembler [50] and TIGR Assembler [51] were used to assemble random sequence data for BV and BAC clones. Gap closure was assisted by a closure editor tool called Cloe that also permits the manual inspection and editing of sequence data. A variety of methods were used to close gaps, including re-sequencing the ends of random clones, transposon assisted sequencing (GPS, New England Biolabs™, Ipswitch, MA, USA) or 'micro-library' construction of single or pooled templates, and conversion of physical gaps to sequence gaps using 'POMP' (pipette optimal multiplex PCR) [52] and or/a 'Genome Walker' kit (Invitrogen™).

### Viral segment-specific PCRs and hybridizations

Primers were developed specific to individual identified GiBV and GfBV viral segment sequences as described in [15]. PCR was performed in a 10 μl solution that included 0.1 μl template DNA, 0.3 μl 50 mM MgCl_2_, 1 μl 10× PCR buffer, 0.2 μl 10 mM dNTPs, 7.9 μl H_2_O, 0.1 μl Platinum Taq (Invitrogen), 0.2 μl F primer (20 pm/μl), and 0.2 μl RC primer (20 pm/μl). The PCR protocol was 94° for 2 minutes; 35 cycles of 94° for 30 s, 58° for 30 s, 72° for 45 s; followed by 72° for 7 minutes. PCR products to be used for hybridizations were purified using a QIAquick PCR purification kit (Qiagen). Segment-specific hybridizations were done as described above for total viral DNA hybridizations.

### Derivation of consensus segment sequences for GiBV and GfBV

Because individual sequence reads could not be associated with individual wasps, a conical consensus sequence was generated for each BV segment using the SliceTools package [53]. At a given position in a conical consensus, all bases with a cumulative quality value within 50% of the highest cumulative quality value were assigned to that position.

### Annotation

A combination of SoftBerry's FGENESH [54] using the honey bee (*A. mellifera*) training set, and the Beijing Genome Institute's BGF [55] trained on the silkmoth (*B. mori*) were used for gene prediction, in addition to the AAT package [56], which allows spliced alignment of proteins to genomic DNA, thereby revealing potential exon-intron boundaries. Gene models from FGENESH were generally accepted except when multiple other sources of information contradicted those models. SignalP [57,58] and tRNAScan-SE [59] were used to predict signal peptides and tRNAs, respectively. Transposable elements were annotated using ProteinRepeatMasker [41] and CENSOR [42].

### Motif analyses

Excision motifs were generated by cutting out a sequence extending 30 bp upstream and downstream from the GCT excision site at the 5' and 3' boundaries of proviral segments and at the GCT circularization site of viral segments for both GiBV and GfBV. No additional alignment was conducted on the sequences. All motifs were visualized using WebLogo [60,61]. Proviral excision motifs were also generated with these sequences using MEME [33], and the resulting motifs were used to search BAC sequences for potential additional proviral segments using MAST [34].

### Comparative genomic analyses

Jaccard orthologous gene clusters between GiBV and GfBV were calculated using Sybil [62]. Syntenic blocks were defined as two or more adjacent orthologous gene clusters, and the results were visualized using Sybil [62]. This information, in addition to conserved location (locus and position within that locus) of proviral segments, was used to define homologous segments between GiBV and GfBV.

### Phylogenetic and compositional analyses

For the phylogenetic analysis, GiBV and GfBV MFS transporter genes were searched against the GenBank non-redundant protein database using BLASTP. The top 18 hits from unique organisms were downloaded from GenBank (*N. vitripennis*, [GenBank:XP_001607065; XP_001602960]; *A. mellifera*, [GenBank:XP_001120868; XP_395522]; *Tribolium castaneum*, [GenBank:XP_973694; XP_973659], [GenBank:XP_966705; XP_966524]; *Anopheles gambiae*, [GenBank:XP_311836]; *Aedes egypti*, [GenBank:XP_001649205]; *Drosophila melanogaster*, [GenBank:NP_611451; NP_524479; CAA73031; XP_001361445]; *Drosophila pseudoobscura*, [GenBank:XP_001358762]; *Mus musculus*, [GenBank:NP_035525; CAC36405]; and *Rattus norvegicus*, [GenBank:NP_062103]). Additionally, the single GfBV MFS transporter gene was searched against *B. mori *expressed sequence tags in GenBank, and the single strong hit (E = e-61; [Genbank:BJ985900]) was downloaded from GenBank and translated. These sequences, in addition to the eight MFS transporters predicted for GiBV and GfBV, were aligned using ClustalW [63], and regions of ambiguous alignment were removed using Seaview [64], resulting in an alignment of 448 amino acids. The phylogenetic analysis was conducted using MrBayes 3.1.2 [65,66], sampling every 1,000 generations for 1 × 10^7 ^generations. The first 50% of generations were discarded as burn-in, and posterior probabilities were calculated from the remaining 501 sampled generations.

For the nucleotide composition analysis, relative trinucleotide frequencies [67] were calculated for all segment, inter-segmental, and flanking sequences. A Euclidean distance matrix was then constructed from those frequencies. The sequences were then clustered using the neighbor-joining algorithm in PAUP* [68] and the resulting tree was visualized with Treeview [69].

### Molecular evolution analyses

All proteins described here for each genome were divided into two sets: those encoded by proviral segments and flanking genes. For each set, BLASTP in WU-BLAST [70] was used to search the GiBV (or Gi) proteins against the GfBV (or Gf) proteins and vice versa. Nucleotide sequences of reciprocal best hit pairs that appeared in syntenic regions (in the same segment for proviral genes and in the same region for flanking genes) were aligned using the cdna_fast_pair method in T-Coffee [71], and pairs with ambiguous or frameshifted alignments were removed. The codeml program in PAML [72,73] was used to calculate dN/dS (Ka/Ks) for the remaining gene pair alignments. Pairs of proviral genes were further analyzed by using codeml in PAML [72,73] to calculate the number of silent and replacement substitutions. The number of silent and replacement polymorphisms within the GiBV shotgun sequence data for these genes with at least 3× coverage were then calculated using previously described methods [15]. The M-K test [36] was then utilized to test gene pairs for evidence of positive or negative selection.

## Abbreviations

BAC: bacterial artificial chromosome; BV: bracovirus; CcBV: *Cotesia congregata *bracovirus; CiBV: *Chelonus inanitus *bracovirus; CsIV: *Campoletis sonorensis *ichnovirus; dN/dS: ratio of non-synonymous to synonymous substitutions; GfBV: *Glyptapanteles flavicoxis *bracovirus; GiBV: *Glyptapanteles indiensis *bracovirus; IV: ichnovirus; MdBV: *Microplitis demolitor *bracovirus; MFS: major facilitator superfamily; M-K: McDonald-Kreitman; PDV: polydnavirus; PTP: protein tyrosine phosphatase; TE: transposable element; WGS: whole genome shotgun.

## Authors' contributions

VN and DEGR conceived the project. VN, CAD, and DEGR coordinated the project. CAD, DEGR, VN, and MJP designed and performed laboratory procedures and experiments. CAD, MCS, JC, and VN designed and performed computational analyses. CAD, VN, and DEGR wrote the manuscript. CAD and BJH conducted genome annotation. JBH, LJT, and KMJ conducted genome closure. RWF reared parasitoids. DWF and HF conducted library construction. All authors read and approved this manuscript.

## Additional data files

The following additional data are available with the online version of this article. Additional data file 1 is a table showing the molecular evolutionary analyses of GiBV and GfBV.

## Supplementary Material

Additional data file 1 Molecular evolutionary analyses of GiBV and GfBV. Click here for file
