# Supplementary material for: Comparative genomics of mutualistic viruses of Glyptapanteles parasitic wasps
Source: Genome Biol. 2008 Dec 30;9(12):R183. doi: 10.1186/gb-2008-9-12-r183 (PMC2646287; doi:10.1186/gb-2008-9-12-r183)
Supplement: Additional data file 1 — Molecular evolutionary analyses of GiBV and GfBV. [file gb-2008-9-12-r183-S1.doc]

**Additional data file 1. Molecular evolutionary analyses of GiBV and GfBV.** The first two columns show the Genbank accession numbers of homologous gene pairs in GiBV and GfBV or Gi and Gf. The second column shows the viral segment the genes occur on (F = flanking DNA). The remaining columns show the number of synonymous (S) and non-synonymous (NS) changes as calculated in PAML [72, 73] for fixed differences and using previously described methods for polymorphisms [15] (ID = insufficient data; NA = not applicable). The ratio of non-synonymous to synonymous fixed differences (w, dN/dS) is also shown as calculated in PAML [72, 73].

| **Gf/GfBV Acc. Number** | **Gi/GiBV Acc. Number** |  | **Fixed Differences** | |  | **Polymorphisms** | |
| --- | --- | --- | --- | --- | --- | --- | --- |
|  |  | **Segment** | **S** | **NS** | **w** | **S** | **NS** |
| ACE75076 | ABK56995 | 1 | 14.5 | 55.5 | 1.74 | 8 | 20 |
| ACE75063 | ABK56997 | 1 | 7.5 | 20.5 | 1.09 | 3 | 6 |
| ACE75077 | ABK56999 | 1 | 4.3 | 21.7 | 2.01 | 2 | 3 |
| ACE75066 | ABK57001 | 1 | 11.5 | 51.5 | 2.23 | 3 | 13 |
| ACE75080 | ABK57003 | 1 | 13 | 38 | 1.09 | 3 | 0 |
| ACE75081 | ABK57005 | 1 | 11.2 | 50.8 | 2.03 | 0 | 2 |
| ACE75091 | ABK57009 | 2 | 21 | 82 | 1.51 | 1 | 0 |
| ACE75092 | ABK57013 | 2 | 11.5 | 67.5 | 2.34 | 1 | 1 |
| ACE75093 | ABK57014 | 3 | 18.3 | 52.7 | 0.97 | ID | ID |
| ACE75094 | ABK57015 | 3 | 19.2 | 43.8 | 0.92 | 6 | 2 |
| ACE75096 | ABK57017 | 3 | 27 | 53 | 0.68 | 10 | 21 |
| ACE75097 | ABK57018 | 3 | 35.5 | 89.5 | 0.96 | 13 | 15 |
| ACE75099 | ABK57020 | 4 | 24.5 | 112.5 | 1.73 | 11 | 15 |
| ACE75100 | ABK57021 | 4 | 13.8 | 41.2 | 0.93 | 0 | 1 |
| ACE75102 | ABK57023 | 4 | 66 | 111 | 0.62 | 13 | 30 |
| ACE75103 | ABK57024 | 5 | 11.5 | 31.5 | 1.11 | 1 | 1 |
| ACE75106 | ABK57027 | 5 | 20.5 | 30.5 | 0.53 | 9 | 2 |
| ACE75111 | ABK57028 | 5 | 18.3 | 43.7 | 0.84 | 4 | 5 |
| ACE75112 | ABK57029 | 5 | 8.5 | 28.5 | 1.20 | 0 | 4 |
| ACE75115 | ABK57032 | 5 | 18.3 | 67.7 | 1.43 | 5 | 10 |
| ACE75124 | ABK57036 | 6 | 14.8 | 52.2 | 1.41 | 3 | 2 |
| ACE75122 | ABK57037 | 6 | 12.3 | 30.7 | 0.92 | 0 | 2 |
| ACE75121 | ABK57038 | 6 | 9.7 | 44.3 | 2.06 | 4 | 9 |
| ACE75125 | ABK57041 | 6 | 18 | 35 | 0.86 | 6 | 11 |
| ACE75126 | ABK57043 | 6 | 91 | 168 | 0.72 | 17 | 33 |
| ACE75228 | ACE75398 | 10 | 10 | 7 | 0.21 | ID | ID |
| ACE75229 | ACE75399 | 10 | 8 | 22 | 0.99 | ID | ID |
| ACE75230 | ACE75400 | 10 | 3 | 19 | 2.06 | ID | ID |
| ACE75231 | ACE75401 | 10 | 13 | 9 | 0.22 | ID | ID |
| ACE75232 | ACE75403 | 10 | 12.8 | 39.2 | 1.06 | ID | ID |
| ACE75234 | ACE75405 | 10 | 12 | 12 | 0.36 | ID | ID |
| ACE75236 | ACE75407 | 10 | 10.5 | 18.5 | 0.64 | ID | ID |
| ACE75239 | ACE75411 | 10 | 11 | 16 | 0.58 | ID | ID |
| ACE75240 | ACE75412 | 10 | 7 | 16 | 0.82 | ID | ID |
| ACE75241 | ACE75413 | 10 | 6 | 17 | 1.09 | ID | ID |
| ACE75250 | ACE75421 | 13 | 8.5 | 17.5 | 0.65 | 3 | 3 |
| ACE75140 | ACE75431 | 14 | 53 | 142 | 1.00 | 8 | 15 |
| ACE75141 | ACE75432 | 14 | 19 | 53 | 0.96 | 7 | 3 |
| ACE75143 | ACE75435 | 15 | 9.5 | 21.5 | 0.75 | 3 | 1 |
| ACE75144 | ACE75436 | 15 | 14.5 | 56.5 | 1.76 | 2 | 4 |
| ACE75146 | ACE75438 | 15 | 8 | 20 | 0.85 | 2 | 4 |
| ACE75150 | ACE75439 | 15 | 10 | 19 | 0.64 | 0 | 1 |
| ACE75151 | ACE75440 | 15 | 7 | 31 | 1.57 | 2 | 0 |
| ACE75154 | ACE75443 | 17 | 43 | 24 | 0.20 | ID | ID |
| ACE75168 | ACE75456 | 20 | 35.2 | 43.8 | 0.42 | 10 | 4 |
| ACE75165 | ACE75459 | 20 | 14.5 | 45.5 | 1.02 | 4 | 5 |
| ACE75174 | ACE75450 | 21 | 7.3 | 22.7 | 1.09 | ID | ID |
| ACE75173 | ACE75451 | 21 | 19.3 | 50.7 | 1.10 | ID | ID |
| ACE75171 | ACE75453 | 21 | 13 | 13 | 0.46 | ID | ID |
| ACE75212 | ACE75302 | 22 | 27.5 | 33.5 | 0.47 | 2 | 2 |
| ACE75214 | ACE75304 | 22 | 18 | 43 | 1.03 | 3 | 1 |
| ACE75215 | ACE75306 | 22 | 11 | 23 | 0.84 | 3 | 3 |
| ACE75218 | ACE75309 | 22 | 22.5 | 69.5 | 1.14 | 4 | 2 |
| ACE75219 | ACE75311 | 23 | 32.7 | 88.3 | 1.03 | 0 | 0 |
| ACE75221 | ACE75315 | 23 | 15.5 | 47.5 | 1.15 | 3 | 4 |
| ACE75222 | ACE75316 | 23 | 11 | 37 | 1.36 | 1 | 7 |
| ACE75206 | ACE75320 | 24 | 29.5 | 23.5 | 0.26 | 7 | 9 |
| ACE75205 | ACE75321 | 24 | 26.5 | 31.5 | 0.40 | 4 | 5 |
| ACE75204 | ACE75322 | 24 | 24.3 | 81.7 | 1.15 | 2 | 2 |
| ACE75203 | ACE75323 | 24 | 55.3 | 48.7 | 0.28 | 6 | 4 |
| ACE75202 | ACE75324 | 24 | 31 | 26 | 0.30 | 8 | 7 |
| ACE75201 | ACE75325 | 24 | 14 | 24 | 0.55 | 2 | 2 |
| ACE75200 | ACE75326 | 24 | 22.4 | 65.6 | 0.87 | 1 | 5 |
| ACE75199 | ACE75327 | 24 | 82.2 | 94.8 | 0.40 | 13 | 6 |
| ACE75198 | ACE75328 | 24 | 33.5 | 58.5 | 0.52 | 3 | 0 |
| ACE75197 | ACE75329 | 24 | 8.5 | 25.5 | 1.30 | 0 | 0 |
| ACE75280 | ACE75364 | 25 | 14 | 45 | 1.31 | 5 | 1 |
| ACE75281 | ACE75369 | 25 | 18 | 26 | 0.57 | 1 | 2 |
| ACE75282 | ACE75370 | 25 | 15.8 | 29.2 | 0.64 | 3 | 2 |
| ACE75283 | ACE75371 | 25 | 31.5 | 94.5 | 1.21 | 0 | 1 |
| ACE75284 | ACE75372 | 25 | 20 | 47 | 0.88 | 0 | 1 |
| ACE75285 | ACE75373 | 25 | 31 | 40 | 0.43 | 5 | 5 |
| ACE75059 | ABK56988 | F | 24 | 13 | 0.23 | NA | NA |
| ACE75060 | ABK56989 | F | 13 | 4 | 0.13 | NA | NA |
| ACE75061 | ABK56990 | F | 41 | 28 | 0.25 | NA | NA |
| ACE75131 | ABK57054 | F | 46.9 | 51.1 | 0.33 | NA | NA |
| ACE75132 | ABK57056 | F | 15 | 4 | 0.10 | NA | NA |
| ACE75133 | ABK57057 | F | 10 | 28 | 1.07 | NA | NA |
| ACE75210 | ACE75299 | F | 44.5 | 101.5 | 0.85 | NA | NA |
| ACE75211 | ACE75301 | F | 11 | 6 | 0.18 | NA | NA |
| ACE75225 | ACE75318 | F | 11.2 | 28.8 | 0.98 | NA | NA |
| ACE75207 | ACE75319 | F | 55.4 | 78.6 | 0.41 | NA | NA |
| ACE75192 | ACE75335 | F | 32.2 | 31.8 | 0.29 | NA | NA |
| ACE75191 | ACE75336 | F | 33 | 8 | 0.09 | NA | NA |
| ACE75190 | ACE75337 | F | 42.8 | 81.2 | 0.64 | NA | NA |
| ACE75189 | ACE75338 | F | 39.5 | 15.5 | 0.14 | NA | NA |
| ACE75188 | ACE75339 | F | 41 | 7 | 0.06 | NA | NA |
| ACE75187 | ACE75340 | F | 35 | 17 | 0.17 | NA | NA |
| ACE75186 | ACE75341 | F | 40.5 | 32.5 | 0.27 | NA | NA |
| ACE75185 | ACE75344 | F | 24 | 38 | 0.56 | NA | NA |
| ACE75184 | ACE75346 | F | 17.2 | 39.8 | 0.82 | NA | NA |
| ACE75183 | ACE75347 | F | 25 | 6 | 0.10 | NA | NA |
| ACE75279 | ACE75363 | F | 11 | 9 | 0.35 | NA | NA |
| ACE75286 | ACE75374 | F | 30 | 1 | 0.01 | NA | NA |
| ACE75287 | ACE75375 | F | 17 | 22 | 0.42 | NA | NA |
| ACE75288 | ACE75376 | F | 17 | 19 | 0.49 | NA | NA |
| ACE75289 | ACE75377 | F | 109 | 16 | 0.06 | NA | NA |
| ACE75290 | ACE75378 | F | 19 | 4 | 0.08 | NA | NA |
| ACE75291 | ACE75379 | F | 12 | 4 | 0.13 | NA | NA |
| ACE75292 | ACE75380 | F | 7 | 0 | 0.00 | NA | NA |
| ACE75293 | ACE75381 | F | 7 | 3 | 0.13 | NA | NA |
| ACE75294 | ACE75382 | F | 7 | 13 | 0.73 | NA | NA |
| ACE75295 | ACE75383 | F | 3 | 2 | 0.20 | NA | NA |
| ACE75296 | ACE75384 | F | 13 | 2 | 0.07 | NA | NA |
| ACE75297 | ACE75385 | F | 31.2 | 17.8 | 0.17 | NA | NA |
| ACE75135 | ACE75387 | F | 2 | 8 | 1.59 | NA | NA |
| ACE75136 | ACE75388 | F | 56 | 120 | 0.66 | NA | NA |
| ACE75137 | ACE75389 | F | 5.5 | 17.5 | 1.04 | NA | NA |
| ACE75138 | ACE75390 | F | 8 | 2 | 0.08 | NA | NA |
| ACE75182 | ACE75447 | F | 42 | 17 | 0.14 | NA | NA |
| ACE75176 | ACE75448 | F | 25 | 2 | 0.03 | NA | NA |
| ACE75175 | ACE75449 | F | 14.2 | 36.8 | 0.95 | NA | NA |
| ACE75259 | ACE75469 | F | 71.2 | 148.8 | 0.74 | NA | NA |
